# Hepatoprotective Effect of *Opuntia robusta* and *Opuntia streptacantha* Fruits against Acetaminophen-Induced Acute Liver Damage

**DOI:** 10.3390/nu8100607

**Published:** 2016-10-04

**Authors:** Herson Antonio González-Ponce, María Consolación Martínez-Saldaña, Ana Rosa Rincón-Sánchez, María Teresa Sumaya-Martínez, Manon Buist-Homan, Klaas Nico Faber, Han Moshage, Fernando Jaramillo-Juárez

**Affiliations:** 1 Department of Physiology and Pharmacology, Basic Science Center, Universidad Autónoma de Aguascalientes, Aguascalientes 20131, Mexico; herson_qfbd@hotmail.com; 2 Department of Morphology, Basic Science Center, Universidad Autónoma de Aguascalientes, Aguascalientes 20131, Mexico; mcmtzsal@correo.uaa.mx; 3 Department of Physiology, University Center of Health Sciences, Universidad de Guadalajara, Guadalajara 44340, Mexico; anarosarincon@yahoo.com.mx; 4 Food Technology Unit, Secretary of Research and Graduate Studies, Universidad Autónoma de Nayarit, Tepic 63160, Mexico; teresumaya@hotmail.com; 5 Department of Gastroenterology and Hepatology, University Medical Center Groningen, University of Groningen, Groningen 9713 GZ, The Netherlands; m.buist-homan@umcg.nl (M.B.-H.); k.n.faber@umcg.nl (K.N.F.); a.j.moshage@umcg.nl (H.M.); 6 Department of Laboratory Medicine, University Medical Center Groningen, University of Groningen, Groningen 9713 GZ, The Netherlands

**Keywords:** *Opuntia* fruits, acetaminophen, acute liver failure, antioxidants, hepatoprotective, nutraceutical

## Abstract

Acetaminophen (APAP)-induced acute liver failure (ALF) is a serious health problem in developed countries. *N*-acetyl-l-cysteine (NAC), the current therapy for APAP-induced ALF, is not always effective, and liver transplantation is often needed. *Opuntia* spp. fruits are an important source of nutrients and contain high levels of bioactive compounds, including antioxidants. The aim of this study was to evaluate the hepatoprotective effect of *Opuntia robusta* and *Opuntia streptacantha* extracts against APAP-induced ALF. In addition, we analyzed the antioxidant activities of these extracts. Fruit extracts (800 mg/kg/day, orally) were given prophylactically to male Wistar rats before intoxication with APAP (500 mg/kg, intraperitoneally). Rat hepatocyte cultures were exposed to 20 mmol/L APAP, and necrosis was assessed by LDH leakage. *Opuntia robusta* had significantly higher levels of antioxidants than *Opuntia streptacantha*. Both extracts significantly attenuated APAP-induced injury markers AST, ALT and ALP and improved liver histology. The *Opuntia* extracts reversed APAP-induced depletion of liver GSH and glycogen stores. In cultured hepatocytes, *Opuntia* extracts significantly reduced leakage of LDH and cell necrosis, both prophylactically and therapeutically. Both extracts appeared to be superior to NAC when used therapeutically. We conclude that *Opuntia* extracts are hepatoprotective and can be used as a nutraceutical to prevent ALF.

## 1. Introduction

Non-steroidal anti-inflammatory drugs (NSAID) are widely used for the alleviation of pain, fever and inflammation. NSAID are the most widely prescribed medications in the world and are used by millions of patients on a daily basis. However, excessive consumption of NSAID has been related to severe side effects caused by oxidative stress, resulting in considerable morbidity and mortality [1,2]. Acetaminophen (APAP), a non-prescription drug, is a safe and effective analgesic and antipyretic drug when used at therapeutic doses [3]. However, an acute or cumulative overdose can cause severe liver injury that may progress to acute liver failure (ALF). In fact, APAP is the most common cause of ALF in developed countries [4,5].

The liver is the main organ involved in the metabolism of APAP. At therapeutic doses, APAP is eliminated via glucuronidation and sulfation reactions. However, at high doses, the conjugation pathways are saturated, and part of the drug is converted by cytochrome P450 2E1 (CYP2E1) to the highly reactive metabolite *N*-acetyl-*p*-benzoquinone imine (NAPQI) that reacts with sulfhydryl groups. Reduced glutathione (GSH) initially traps NAPQI, and the GSH adduct is excreted. However, when GSH is depleted, NAPQI reacts with cellular proteins, including a number of mitochondrial proteins, to form NAPQI adducts. Consequences of this process are the inhibition of mitochondrial respiration and ATP depletion, as well as mitochondrial oxidative stress [6,7,8]. 

This results in increased susceptibility to liver injury by reactive oxygen species (ROS), including hydrogen peroxide (H_2_O_2_), superoxide anions (O_2_^•−^) and hydroxyl radicals (·OH). In addition to reducing the GSH level, the APAP overdose also reduces the antioxidant enzyme activities, increases lipid peroxidation and causes hepatic DNA fragmentation, which ultimately leads to cellular necrosis [9,10].

Currently, the treatment of choice for APAP overdose is *N*-acetyl-l-cysteine, a precursor of intracellular cysteine and GSH that counteracts the depletion of GSH and allows the excretion of NAPQI as the GSH-adduct. This reduces oxidative stress and, consequently, liver injury [11]. Unfortunately, *N*-acetyl-l-cysteine is not always effective, and there is an urgent need for more effective interventions. 

Medicinal benefits from plants have been recognized for centuries. Vegetables and fruits are very important in human nutrition and as sources of phytochemicals that reduce disease risks, like oxidative stress, inflammation and DNA damage [12,13]. The protective effects of diets rich in fruits and vegetables are not only due to fibers, vitamins and minerals, but also to secondary metabolites of plant products [14]. In recent years, many antioxidant compounds, such as vitamins, pigments and phenolic phytochemicals from fruits, vegetables and herbs, have received special attention due to their protective actions against oxidative damage and genotoxicity [15,16].

Cactus (*Opuntia* spp.) is used as a common vegetable and medicinal plant on the American continent. There are about 200 recognized species of *Opuntia*, and at least 84 are found in México [17,18]. Cactus pears are sweet edible fruits from the cactus (*Opuntia* spp.) that belong to the Cactaceae family [19]. These fruits have been used in traditional medicine for the treatment of several diseases [20] and contain a wide variety of trace elements, sugars and other bioactive compounds, such as betalains, carotenoids, ascorbic acid, flavonoids and other phenolic compounds [21]. Cactus pear fruits are now recognized as a rich source of nutritional compounds with health-promoting activities, including antioxidant [22,23,24,25,26], neuroprotective, anti-inflammatory, cardioprotective, anti-diabetic [27], anti-clastogenic [28] and anti-genotoxic actions [16]. In addition, they have protective effects on erythrocyte membranes [29] and on acute gastric lesions [30], and they improve platelet function [31] and cancer chemoprevention [18]. Interestingly, APAP-induced liver injury is one of the most widely-used models to evaluate the hepatoprotective potential of natural products [32].

The aim of this study was to investigate the hepatoprotective effect of *Opuntia robusta* and *Opuntia streptacantha* fruits from a semi-arid region of Mexico in a model of APAP-induced liver injury and to perform an initial characterization of the main bioactive compounds in these fruits.

## 2. Experimental Procedures

### 2.1. Chemicals and Materials

Stock solutions of acetaminophen (APAP, 2 mol/L), reduced glutathione (GSH, 50 mmol/L) and *N*-acetyl-l-cysteine (NAC, 1 mol/L) were prepared in the appropriate solvents (phosphate-buffered saline for in vivo experiments and culture medium for the in vitro experiments). All reagents were from Sigma Aldrich, St. Louis, MO, USA. Sytox green nucleic acid stain, gentamycin, William’s E medium and fetal calf serum were obtained from Invitrogen (Breda, The Netherlands); penicillin-streptomycin-fungizone (PSF) was from Lonza (Verviers, Belgium); microplate readers Biotek PowerWave XS and Biotek Synergy HT were from BioTek Instruments Inc. (Winooski, VT, USA).

### 2.2. Animals

Adult male Wistar rats (200–250 g) were used for the in vivo and in vitro studies. The animals were kept in polypropylene cages at room temperature (25 ± 2 °C) with food and water ad libitum. Experiments were approved by and performed following the guidelines of the local Committee for Care and Use of laboratory animals (Permission No. 6415A of the Committee for Care and Use of laboratory animals of the University of Groningen and Mexican governmental guideline NOM 033 ZOO 1995).

### 2.3. Plant Materials and Extracts Preparation

Ripe fruits of *Opuntia robusta* and *Opuntia streptacantha* were collected from randomly-selected plants of the same species in the semi-arid region of Aguascalientes, México (21°46′55.86″ N, 102°6′16.08″ O, and 1994 meters above sea level). The juice of each batch of peeled fruits was extracted and mixed in a single procedure using a Braun J500 juice extractor (Braun GmbH, Taunus, Germany). The collected juice of each batch was aliquoted into 50-mL dark tubes, centrifuged at 5000 rpm for 15 min at 4 °C, filtered through Whatman 40 filter paper (8-μm pore size), stored at −80 °C and lyophilized in a Labconco FreeZone Freeze Dry System (Labconco Corp, Kansas City, MO, USA) [26,28]. Extracts of one batch were used for the entire study. Lyophilized extracts were reconstituted with 50 mL of deionized water. 

### 2.4. Determination of the Main Bioactive Compounds of Fruit Extracts

The reconstituted extract of each *Opuntia* species was clarified (12,000× *g* for 15 min at 15 °C) and used to determine the bioactive compounds. Flavonoids were quantified by the colorimetric method of Zhishen et al. [33], and values were expressed as µg quercetin equivalents/mL. The total content of betalains (betacyanins and betaxanthins) was determined according to the methods described by Stintzing et al. [34] and Sumaya-Martínez et al. [26], and results were expressed as mg of betacyanin or betaxanthin equivalents/L. Ascorbic acid content in the extracts was determined by the method of Dürüst et al. [35], and the results were expressed as mg ascorbic acid equivalents/L. The total phenolic content was determined by the Folin-Ciocalteu method described by Georgé et al. [36] and was expressed as mg gallic acid equivalents/L. All measurements were performed in triplicate on a Biotek PowerWave XS microplate reader.

### 2.5. Determination of Free Radical Scavenging and Chelating Activities

The antioxidant activity of the *Opuntia* fruit extracts was determined by different methods. The DPPH (2,2-diphenyl-1-picrylhydrazyl) method was performed according to Morales and Jimenez [37], and the antioxidant activity was expressed as mmol Trolox equivalents/L. The ABTS (2,2′-azino-bis(3-ethylbenzothiazoline-6-sulphonic acid)) method was performed according to Re et al. [38] and Kuskoski et al. [39], and the results were expressed as mg ascorbic acid (AA)/100 mL. FRAP (ferric reducing antioxidant power) was determined as described by Hinneburg et al. [40], and the results were expressed as mg ascorbic acid (AA)/100 mL. The assay to determine the chelating activity was performed as described by Gulcin et al. [41] and was expressed as mol EDTA equivalent/L. All measurements were performed in triplicate on a Biotek PowerWave XS microplate reader. The ORAC (oxygen radical absorbance capacity) was determined as described by Huang [42], and the results were expressed as mmol Trolox equivalents/L. This assay was performed in triplicate on a Biotek Synergy HT microplate reader.

### 2.6. Rat Hepatocyte Isolation

Hepatocytes were isolated from male Wistar rats by two-step collagenase perfusion as described by Conde de la Rosa et al. [43] and Vrenken et al. [44]. The cell viability was determined by trypan blue exclusion assay and was always higher than 85%. After isolation, the hepatocytes were plated on 6-well coated plates in William’s E medium supplemented with 50 μg/mL gentamycin, 1% penicillin-streptomycin-fungizone and 10% fetal calf serum. Cells were cultured for 4 h to attach in a humidified incubator at 37 °C and 5% CO_2_.

### 2.7. Experimental Design

#### 2.7.1. In Vivo Study

Animals were randomly divided into seven groups (*n* = 12): (1) control (C); (2) acetaminophen treated (APAP); (3) *Opuntia robusta* treated (*Or*); (4) *Opuntia streptacantha* treated (*Os*); (5) *Or* + APAP treated; (6) *Os* + APAP treated; and (7) GSH + APAP treated. Rats were pretreated with daily oral doses of cactus extract (800 mg/kg), according to the protocol of Kim et al. [30] with some modifications) or GSH (50 mg/kg, intraperitoneally) for five days before APAP treatment (500 mg/kg intraperitoneally; single dose). Samples of blood and liver tissue were collected 4 h after APAP intoxication for assessment of liver damage markers [45]. Some animals were sacrificed 24 h after APAP intoxication, and liver tissue was obtained for histological evaluation.

Liver damage was assessed by measurement of alanine aminotransferase (ALT), aspartate aminotransferase (AST) and alkaline phosphatase (ALP) serum activities using a commercial spectrophotometrical method (SPINREACT, Girona, Spain). The values represent the mean of three measurements (±standard deviation) and are expressed as IU/L.

The GSH content in liver tissue homogenates was determined according to Hissin and Hilf [46], using *o*-phtaldehyde (OPT) as the fluorescent reagent. The fluorescence intensity was measured at 420 nm using 350 nm as the excitation wavelength, using a Luminescence Spectrophotometer (Model LS-50B, PerkinElmer Inc., Waltham, MA, USA).

For histological studies, animals were anesthetized with sodium pentobarbital and perfused via the portal vein with saline solution (sodium chloride 0.9%), containing 0.5% heparin and 0.1% procaine and fixed in situ with neutral formalin (10%). The hepatic tissue was embedded in paraffin blocks, and sections of 5 µm were prepared with a microtome (Leica RM2125RT). The sections were stained with periodic acid Schiff (PAS) reagent. Liver tissue images were captured using a Carl Zeiss microscope (Axioskope 40) and processed using Image-Pro Plus software.

#### 2.7.2. In Vitro Study

After the attachment period of 4 h, the medium was changed for medium without fetal calf serum. Monolayer cultures were exposed to a toxic dose of acetaminophen (20 mmol/L). A single dose of 125 μL (8% *v*/*v*) of *Opuntia streptacantha* or *Opuntia robusta* reconstituted extract or 5 mmol/L *N*-acetyl-l-cysteine (NAC) was added 30 min before (prophylactic regimen) or 30 min, 1, 2, 4 and 8 h after the addition of acetaminophen (therapeutic regimen).

To quantitatively assess hepatocyte injury, LDH leakage into medium was evaluated. LDH activity was measured spectrophotometrically at 340 nm by determining NADH oxidation in the presence of sodium pyruvate in a microplate reader (BioTek EL808) for 30 min at 37 °C and expressed as fold induction versus control values [47]. Hepatocyte necrosis was also assessed by Sytox Green staining to confirm the LDH leakage results. Sytox green binds to nuclear DNA, but can only enter cells with ruptured plasma membranes, as occurs in necrotic, but not in apoptotic cell death. Necrosis of hepatocytes was determined by incubation for 15 min with Sytox green (1:40,000) nucleic acid stain as described by Woudenberg-Vrenken et al. [48]. Necrosis was quantified by counting fluorescent nuclei and the total number of cells in three randomly chosen high power fields. Fluorescent nuclei were visualized using a Leica microscope DMI 6000B at 450–490 nm.

### 2.8. Statistical Analysis of Data

For the analysis of the components and antioxidant activities of cactus fruit extracts, the unpaired *t*-test was carried out with a confidence level of 99%. The analysis of biochemical results was done using the one-way ANOVA test, and the means of the different experimental groups were compared using the Tukey test with a confidence level of 95%. GraphPad Prism 5 software was used for the statistical analysis (La Jolla, CA, USA).

## 3. Results

### 3.1. Yields of the Juice Extraction Method

**Figure 1:**
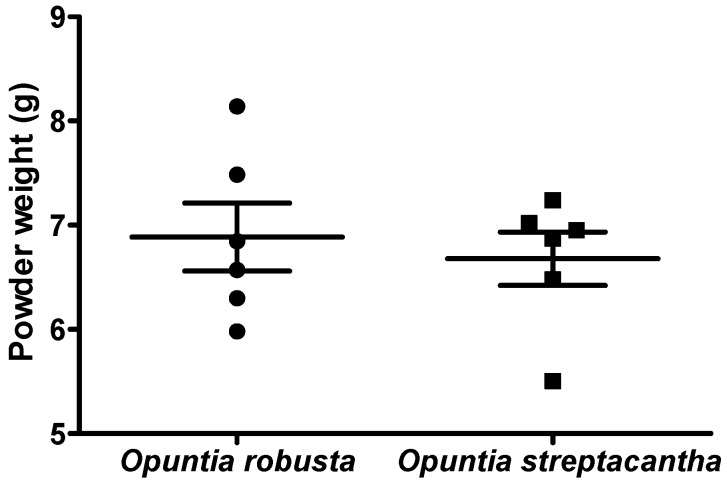
Yield (gram powder) obtained after lyophilization of 50 mL of juice. Each scatter column represents the mean of six samples ± SEM. *p* > 0.05.

Depending on the size and ripeness stage, approximately three fruits of *Opuntia robusta* and six fruits of *Opuntia streptacantha* were used to obtain 50 mL of juice extract. After lyophilization of 50 mL of each fruit juice, 6.89 ± 0.80 g of *Opuntia robusta* and 6.68 ± 0.63 g of *Opuntia streptacantha* powder were obtained (Figure 1). The difference in yield between the two cactus species was not statistically significant (*p* > 0.05). For the in vivo experiments, a dose of 800 mg/kg in rats (200–250 g b.w.) corresponded to approximately 1.5 mL of the reconstituted extract. For the in vitro experiments, a dose of 8% *v*/*v* in primary rat hepatocytes corresponded to approximately 125 µL of the reconstituted extract.

### 3.2. Bioactive Compounds of Cactus Pear Fruit Extracts

The extracts of *Opuntia robusta* and *Opuntia streptacantha* ripe fruits contain a high quantity of bioactive compounds. *Opuntia robusta* had a significantly higher amount of flavonoids, ascorbic acid and total phenolic compounds than *Opuntia streptacantha* (Table 1). *Opuntia robusta* contained a significantly higher amount of betacyanin than *Opuntia streptacantha*. In addition, both *Opuntia* fruit extracts had more betacyanin than betaxanthin, causing the red-purple color of the fruits (Table 2). Similar values of these compounds have been reported in other *Opuntia* species [26,34].

### 3.3. Free Radical Scavenging and Chelating Activities of Opuntia Extracts

Both *Opuntia* species demonstrated free radical scavenging capacity, but to different extents (Table 3). *Opuntia robusta* had superior antioxidant activity compared to *Opuntia streptacantha*, which might be due to its chemical composition since *Opuntia robusta* contains higher amounts of bioactive compounds (Table 1 and Table 2). On the other hand, extracts of *Opuntia streptacantha* contained more chelating activity than *Opuntia robusta* (Table 4).

### 3.4. In Vivo Experiments

The plasma levels of transaminases and alkaline phosphatase, as well as the GSH content in liver homogenates of experimental animals 4 h after APAP intoxication are shown in Table 5. APAP treatment significantly increased plasma levels of transaminases and ALP compared to control animals (*p* < 0.05). The prophylactic administration of both *Opuntia* fruit extracts (*Or* + APAP and *Os* + APAP) in APAP-treated animals significantly decreased all markers of liver cell injury compared to APAP-intoxicated animals (*p* < 0.05). The extracts alone did not cause any changes in these plasma markers (Table 5). GSH appeared to be less effective in attenuating liver damage compared to the *Opuntia* extracts (Table 5).

GSH was almost completely depleted after APAP overdose. *Opuntia robusta* extract completely and *Opuntia streptacantha* extract partially prevented the depletion of GSH induced by APAP treatment. *Opuntia* extracts alone did not change hepatic GSH levels. GSH administration partially prevented the depletion of hepatic GSH levels, comparable to *Opuntia streptacantha*. Thus, *Opuntia robusta* had superior protective effects than *Opuntia streptacantha* in acute APAP intoxication.

**Figure 2:**
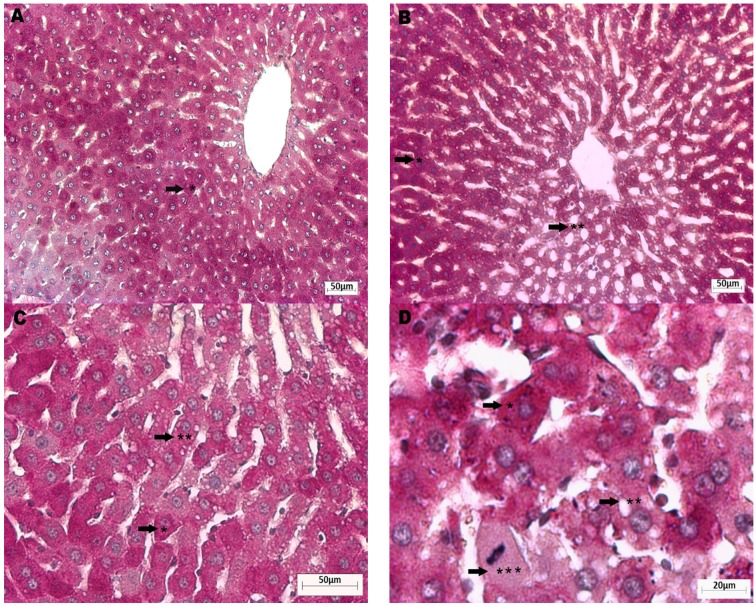
Histopathological images of liver tissue of non-treated control animals and APAP-treated animals. (**A**) Control: normal morphology in all zones of the hepatic acinus with positive PAS staining, indicating glycogen stores (black arrow *); magnification 100×; (**B**) APAP group: intense cytoplasmic vacuolation of hepatocytes nearest to the central vein (Zones II and III; black arrow **); magnification 100×; (**C**) PAS staining of APAP group indicating depletion of cytoplasmic glycogen stores and vacuolation of the hepatocytes near the central vein (black arrow **); magnification 200×; (**D**) APAP group: focal necrosis of hepatocytes (black arrow ***); magnification 400×.

**Figure 3:**
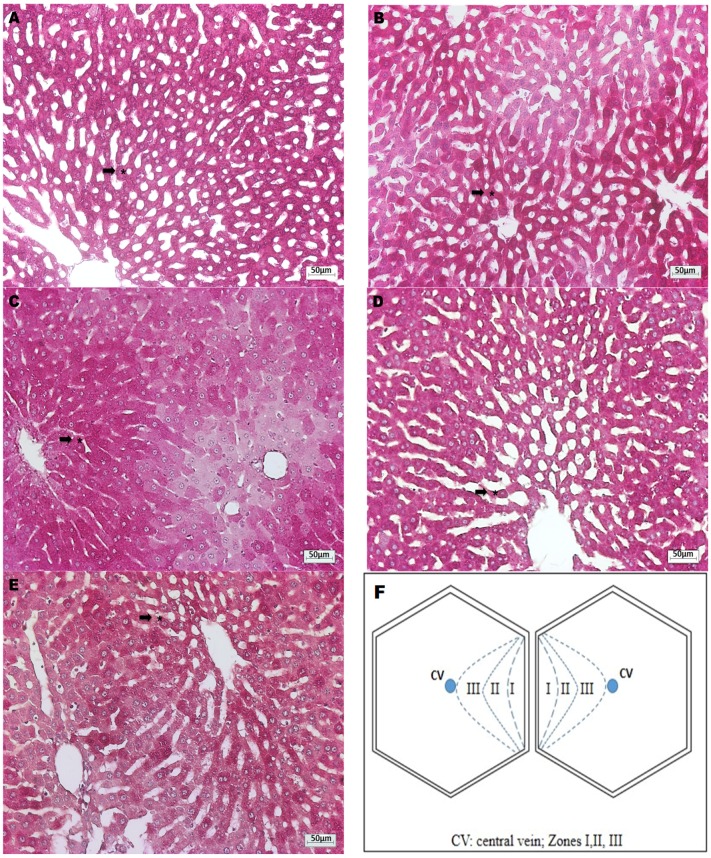
Histopathological images of liver tissue of APAP-intoxicated rats, prophylactically treated with *Opuntia* extracts. (**A**) *Opuntia robusta*-treated group and (**B**) *Opuntia streptacantha*-treated group: hepatocytes of Zones II and III to hepatic acinus showed normal morphology and PAS positive reaction (black arrow *); magnification 100×; (**C**) *Opuntia robusta* + APAP group; (**D**) *Opuntia streptacantha* + APAP; and (**E**) GSH + APAP group: normal morphology and PAS positive staining of pericentral (Zones II and III) hepatocytes (black arrow *); magnification 100×; (**F**) Zones of the hepatic acinus.

In the APAP group, an extensive hydropic vacuolation was observed (Figure 2B), as well as glycogen depletion (Figure 2C) and focal necrosis of cells with pyknotic nuclei (Figure 2D) of the hepatocytes nearest to the central vein compared to the control group (Figure 2A). *Opuntia* extracts alone did not cause morphological changes, glycogen depletion or necrosis (Figure 3A,B). Both *Opuntia* extracts attenuated the histological changes induced by APAP alone and maintained cytoplasmic glycogen stores, without signs of vacuolation or necrosis in the hepatic acinus (Figure 3C,D). GSH supplementation also prevented vacuolation and necrosis, although some hepatocytes lost glycogen stores (Figure 3E).

### 3.5. In Vitro Experiments

**Figure 4:**
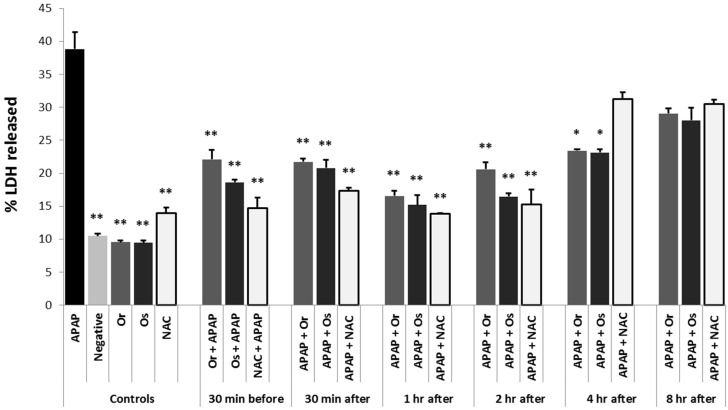
*Opuntia* extracts protect against APAP-induced toxicity in primary cultures of rat hepatocytes. Cell toxicity is represented as LDH released into medium 24 h after the addition of 20 mmol/L APAP. *Opuntia* extracts and NAC were added at different time points before and after APAP intoxication. *Or*, *Opuntia robusta*; *Os*, *Opuntia streptacantha*; APAP, acetaminophen; NAC, *N*-acetyl-l-cysteine. Results are expressed as fold-increase relative to control group; * *p* < 0.05, ** *p* < 0.01 compared to the APAP group.

Both *Opuntia* extracts protected the hepatocytes against APAP-induced cell necrosis when added prior to APAP (prophylactic regimen), as shown in Figure 4. NAC was also protective, but tended to be less potent than the *Opuntia* extracts. In the therapeutic regimen, both *Opuntia* extracts protected against APAP-induced toxicity when added up to 4 h after APAP, whereas NAC was only protective when added up to 1–2 h after APAP (Figure 4). 

**Figure 5:**
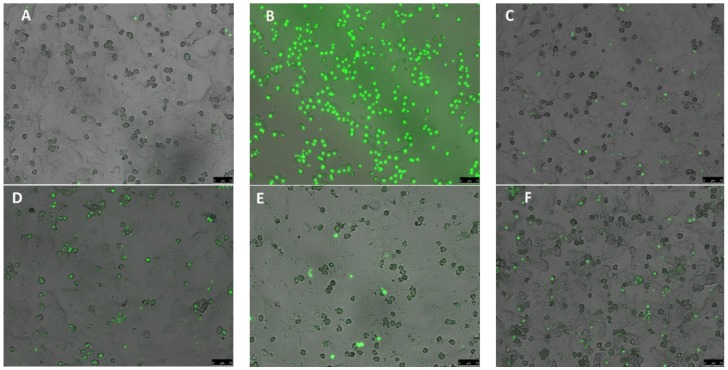

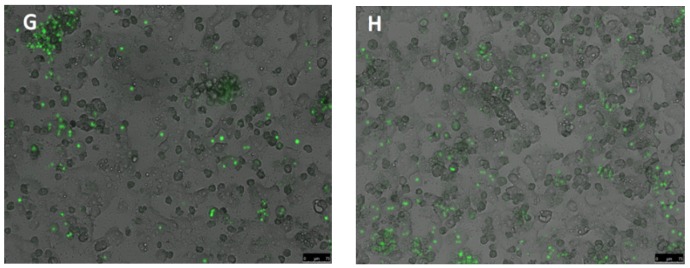
*Opuntia* extracts protect against APAP-induced necrotic cell death in primary cultures of rat hepatocytes. Cell toxicity was determined using the Sytox green fluorescent dye for necrotic cells. *Opuntia* extracts and NAC were added 30 min prior to APAP. Hepatocytes were exposed to 20 mmol/L APAP for 24 h, and subsequently, Sytox green was added. Micrographs were taken 15 min after addition of Sytox green. Different groups are explained in the lower-right panel. Magnification 100×. (**A**) Control; (**B**) APAP; (**C**) *O. robusta*; (**D**) *O. streptacantha*; (**E**) NAC; (**F**) *O. robusta* + APAP; (**G**) *O. streptacantha* + APAP; (**H**) NAC + APAP.

To confirm the results obtained by determining the LDH release, we also used Sytox green as a fluorescent dye for necrotic cells. APAP (20 mmol/L) induced significant cell necrosis in primary cultures of rat hepatocytes (Figure 5B) in comparison to the Control (Figure 5A), which was almost completely abolished by the *Opuntia* extracts (Figure 5F,G). NAC (Figure 5H) showed a similar protective effect as the *Opuntia* extracts. These results confirm the data obtained with the LDH determinations.

## 4. Discussion

Natural compounds have a huge structural diversity and many biological activities, thus offering ample opportunities to identify novel compounds for the treatment of different diseases [49,50]. The presence or absence of many bioactive compounds in leaves, fruits, roots, seeds and other natural subproducts depends on geographical and environmental factors, such as humidity, temperature, season, pollution, altitude, etc. Therefore, it is very difficult to standardize the composition of natural products, but it is acceptable to link their therapeutic benefits to the presence and concentration of specific compounds in the extracts used [51]. We studied two *Opuntia* species that are widely distributed in the central semi-arid regions of Mexico [52]. The fruits contained a large quantity of the most important phytochemical compounds with proven therapeutic activity as reported by Vinson et al. [53] and Coria Cayupán et al. [24]. A chemical characterization of the main bioactive compounds present in these two species of *Opuntia* fruits from different areas has been performed previously by different authors, and the results showed that the main components of them are mostly betalains, specifically betacyanins; flavonoids and phenolic compounds that apparently are responsible for their biologic activity [20,23].

Bioflavonoids are widely distributed in fruits and vegetables and have multiple biological effects, including free radical scavenging activity and chelation of metal ions [54]. It is well known that flavonoid effects are related to their chemical structure. This is especially true for flavonols, such as quercetin, which represents the most abundant dietary flavonoid. Mechanisms of antioxidant action include the suppression of reactive oxygen species (ROS) formation either by inhibition of ROS-generating enzymes; the chelation of trace elements that are involved in free radical generation; or by the induction of antioxidant defenses. These abilities are intimately related to the oxidation/reduction potential and the activation energy for electron transfer of the substance [55,56,57]. Several therapeutic effects of flavonoids have been linked to their antioxidant capacity, e.g., the inhibition of inflammation [58] and lipoperoxidation [59], as well as nephroprotective [60,61], neuroprotective [62] and hepatoprotective activities [63]. In addition, there is increasing evidence that polyphenols protect cellular constituents against oxidative damage and, therefore, limit the risk of chronic diseases associated with oxidative stress [64]. 

The fruit extracts of both *Opuntia* species investigated in this study contain high concentrations of betalains. The chemical structure of these pigments is derived from betalamic acid, and depending on the structures added to the main structure, they give rise to betacyanins and betaxanthins [65]. These bioactive compounds are natural antioxidants with a high radical scavenging potential [66]. The betanin molecule includes phenolic and cyclic amine groups, which are potent electron donors that endow betanin with an exceptionally high free radical scavenging ability [67]. Studies have investigated the capacity of betalains, mainly betanin, to scavenge free radicals in vitro, and this capacity is even higher than that of vitamin C [34,68]. Betanin has also been reported to inhibit cancer cell proliferation in vitro and in vivo [18], and it protects against acute lung injury and gastric lesions [30,69].

At high doses, the metabolism of APAP leads to the generation of the highly-reactive metabolite NAPQI, which leads to GSH depletion and the subsequent reaction of NAPQI with cellular proteins and lipids to form APAP adducts. Mitochondria are one of the most important targets of NAPQI, resulting in ATP depletion, oxidative stress and ultimately hepatocyte necrosis [7,8]. It has been reported that the hepatoprotective effect of some natural products is related to their antioxidant capacity to prevent liver cell damage or death, both prophylactically and therapeutically [70,71]. 

Our results suggest that the frequent consumption of *Opuntia robusta* and *Opuntia streptacantha* provides many bioactive compounds with antioxidant activity to counteract the cellular oxidative damage caused by APAP acute intoxication. Therapeutic treatment more closely resembles the clinical situation of APAP intoxication; however, the primary goal of this study was to demonstrate the protective effect of *Opuntia* extracts in APAP intoxication. Although we demonstrate in vitro the value of therapeutic treatment, this needs to be confirmed in vivo, as well. Animal studies have revealed the promising in vivo therapeutic value of antioxidants on liver diseases. Furthermore, APAP is a model of severe oxidative stress, and in many liver diseases, oxidative stress precedes or aggravates existing liver diseases (e.g., in non-alcoholic liver diseases). In fact, oxidative stress is considered as a common mechanism of liver injury in many (chronic) liver diseases, and the application of antioxidants is a rational strategy to prevent or ameliorate liver diseases involving oxidative stress [72]. Therefore, there is certainly value in the prophylactic use of *Opuntia* extracts as anti-oxidants, and we are currently testing *Opuntia* extracts in other models of (oxidative) liver damage. 

The CYP2E1 enzyme is considered to be the main enzyme responsible for APAP biotransformation, and this enzyme is predominantly expressed in the centrilobular region [73]. In line with this, we observed the most prominent histological damage in the centrilobular area (Zones II/III of the hepatic acinus). Our histological data are also consistent with necrotic hepatocyte loss. This is confirmed by our in vitro data that clearly show that necrosis (Sytox green, LDH leakage) is the predominant mode of cell death in APAP intoxication. The *Opuntia* extracts are still protective when added up to 4 h after APAP intoxication, and in this respect, they are superior to NAC, the currently-used therapy for ALF induced by APAP. The reason for this might be that the *Opuntia* extracts contain a multitude of components that counteract oxidative damage via different mechanisms. Future studies should identify the components of *Opuntia* extracts that contribute to this protective effect. 

In summary, we provide evidence that both *Opuntia* fruit extracts contain many bioactive compounds with antioxidant activity to counteract the oxidative damage caused by APAP. Our results also suggest that the daily ingestion of *Opuntia streptacantha* and *Opuntia robusta* fruit extracts at the indicated doses can increase liver detoxification and could be used as a dietary supplement to prevent APAP-induced acute liver failure. Finally, our results also suggest that the *Opuntia* extracts can be considered for other oxidative stress-related liver diseases like (non-)alcoholic fatty liver diseases.

## Figures and Tables

**Table 1:** Total amount of bioactive compounds in the *Opuntia* fruit extracts.

Fruit	Flavonoids (µg eq. Quercetin/mL)	Ascorbic Acid (mg eq. Ascorbic Acid/L)	Total Phenolic Compounds (mg eq. Gallic Acid/L)
*Opuntia robusta*	89.19 ± 2.84 *	328.83 ± 28.47 *	573.73 ± 24.99 *
*Opuntia streptacantha*	54.48 ± 0.93	65.86 ± 12.33	343.12 ± 9.72

Values are the mean of three measurements ± SD. *Opuntia robusta* vs. *Opuntia streptacantha* * *p* < 0.01. eq.: equivalent.

**Table 2:** Betacyanin and betaxanthin content and total amount of betalains in the two *Opuntia* fruit extracts.

Fruit	Betacyanin (mg eq. Betacyanin/L)	Betaxanthin (mg eq. Betaxanthin/L)	Total Betalains (mg eq. Betalains/L)
*Opuntia robusta*	333.27 ± 11.46 *	133.66 ± 4.83 *	466.93 ± 16.29 *
*Opuntia streptacantha*	87.24 ± 1.54	36.47 ± 1.07	123.70 ± 2.61

Values are the mean of three measurements ± SD. *Opuntia robusta* vs. *Opuntia streptacantha* * *p* < 0.01.

**Table 3:** Free radical scavenging activity of *Opuntia* fruit extracts.

Fruit	DPPH (mmol eq. Trolox^®^/L)	FRAP (mg eq. Ascorbic Acid/100 mL)	ABTS (mg eq. Ascorbic Acid/100 mL)	ORAC (mmol eq. Trolox^®^/L)
*Opuntia robusta*	5.77 ± 0.33 *	73.24 ± 3 *	92.62 ± 5 *	41.78 ± 1.89 *
*Opuntia streptacantha*	1.31 ± 0.94	28.82 ± 2	61.69 ± 3	31.42 ± 0.43

DPPH, 2,2-diphenyl-1-picrylhydrazyl; FRAP, ferric reducing antioxidant power; ABST, 2,2′-azino-bis(3-ethylbenzothiazoline-6-sulphonic acid); ORAC, oxygen radical absorbance capacity. Values are the mean of three measurements ± SD. *Opuntia robusta* vs. *Opuntia streptacantha* * *p* < 0.01.

**Table 4:** Chelating activity of *Opuntia* fruit extracts.

Fruit	Ferrous Ion Scavenging (mol eq. EDTA/L)
*Opuntia robusta*	3.69 ± 0.9
*Opuntia streptacantha*	6.09 ± 0.8 *

Values are the mean of three measurements ± SD. *Opuntia robusta* vs. *Opuntia streptacantha* * *p* < 0.01.

**Table 5:** Effect of *Opuntia* fruit extracts on acetaminophen-induced acute liver injury. Levels of transaminases in plasma and reduced glutathione in liver homogenates.

Group	ALT (IU/L)	AST (IU/L)	ALP (IU/L)	GSH (µg/g)
Control	37.9 ± 0.7 *	79.5 ± 4.2 *	319 ± 15.4 *	1797 ± 28 *
APAP	82.4 ± 8.8	320 ± 48.0	512 ± 36.6	198 ± 4
*Or*	37.3 ± 3.8 *	75.2 ± 2.8 *	285 ± 36.2 *	1709 ± 23 *
*Os*	39.9 ± 3.3 *	84.5 ± 5.8 *	251 ± 35.2 *	1519 ± 101 *
*Or* + APAP	41.9 ± 3.3 *	129 ± 10.3 *	246 ± 11.2 *	1608 ± 31 *
*Os* + APAP	58.1 ± 6.1 *	151 ± 33.3 *	459 ± 28.6	666 ± 47 *
GSH + APAP	69.8 ± 5.5	289 ± 42.2	309 ± 15.4 *	604 ± 26 *

APAP: acetaminophen; *Or*: *Opuntia robusta*; *Os*: *Opuntia streptacantha*; GSH: reduced glutathione. Values are the mean of six measurements (±SEM). * *p* < 0.05 regarding the APAP group.

## Abbreviations

AA	Ascorbic acid
ABTS	2,2′-Azino-bis(3-ethylbenzothiazoline-6-sulphonic acid)
ALF	Acute liver failure
ALP	Alkaline phosphatase
ALT	Alanine aminotransferase
ANOVA	Analysis of variance
APAP	Acetaminophen
AST	Aspartate aminotransferase
CAT	Catalase
Cu/Zn-SOD	Copper/zinc-superoxide dismutase
CYP450	Cytochrome P-450
CYP2E1	Cytochrome P-450 isoform 2E1
DNA	Deoxyribonucleic acid
DPPH	2,2-Diphenyl-1-picrylhydrazyl
EDTA	Ethylenediaminetetraacetic acid
FRAP	Ferric reducing antioxidant power
GPx	Glutathione peroxidase
GSH	Reduced glutathione
LDH	Lactate dehydrogenase
Mn-SOD	Manganese-superoxide dismutase
NAC	*N*-acetyl-l-cysteine
NAPQI	*N*-acetyl-*p*-benzoquinone imine
NSAID	Non-steroidal anti-inflammatory drugs
OPT	*o*-phtaldehyde
ORAC	Oxygen radical absorbance capacity
PAS	Periodic acid Schiff
PSF	Penicillin-streptomycin-fungizone
ROS	Reactive oxygen species
SEM	Standard error of the mean
SD	Standard deviation

## References

[1] Vonkeman H.E., van de Laar M.A. (2010). Nonsteroidal Anti-Inflammatory Drugs: Adverse Effects and Their Prevention. Semin. Arthritis Rheum..

[2] Jones R. (2001). Nonsteroidal anti-inflammatory drug prescribing: Past, present, and future. Am. J. Med..

[3] Rumack B.H. (2004). Acetaminophen misconceptions. Hepatology.

[4] Jaeschke H., Bajt M.L. (2005). Intracellular signaling mechanisms of acetaminophen-induced liver cell death. Toxicol. Sci..

[5] Craig D.G.N., Lee A., Hayes P.C., Simpson K.J. (2010). The current management of acute liver failure. Aliment. Pharmacol. Ther..

[6] Jaeschke H., McGill M.R., Williams C.D., Ramachandran A. (2011). Current issues with acetaminophen hepatotoxicity—A clinically relevant model to test the efficacy of natural products. Life Sci..

[7] McGill M.R., Jaeschke H. (2013). Metabolism and disposition of acetaminophen: Recent advances in relation to hepatotoxicity and diagnosis. Pharm. Res..

[8] Jaeschke H., Knight T.R., Bajt M.L. (2003). The role of oxidant stress and reactive nitrogen species in acetaminophen hepatotoxicity. Toxicol. Lett..

[9] Arai T., Koyama M., Kitamura D., Mizuta R. (2014). Acrolein, a highly toxic aldehyde generated under oxidative stress in vivo, aggravates the mouse liver damage after acetaminophen overdose. Biomed. Res..

[10] Das J., Ghosh J., Manna P., Sil P.C. (2010). Acetaminophen induced acute liver failure via oxidative stress and JNK activation: Protective role of taurine by the suppression of cytochrome P450 2E1. Free Radic. Res..

[11] Mahmoodi M., Soleimani Mehranjani M., Shariatzadeh S.M.A., Eimani H., Shahverdi A. (2015). *N*-acetylcysteine improves function and follicular survival in mice ovarian grafts through inhibition of oxidative stress. Reprod. Biomed. Online.

[12] Boeing H., Bechthold A., Bub A., Ellinger S., Haller D., Kroke A., Watzl B. (2012). Critical review: Vegetables and fruit in the prevention of chronic diseases. Eur. J. Nutr..

[13] Poljsak B., Šuput D., Milisav I. (2013). Achieving the Balance between ROS and Antioxidants: When to Use the Synthetic Antioxidants. Oxid. Med. Cell. Longev..

[14] Crozier A., Del Rio D., Clifford M.N. (2010). Bioavailability of dietary flavonoids and phenolic compounds. Mol. Asp. Med..

[15] Heber D. (2004). Vegetables, fruits and phytoestrogens in the prevention of diseases. J. Postgrad. Med..

[16] Brahmi D., Bouaziz C., Ayed Y., Ben Mansour H., Zourgui L., Bacha H. (2011). Chemopreventive effect of cactus *Opuntia ficus* indica on oxidative stress and genotoxicity of aflatoxin B1. Nutr. Metab. (Lond.).

[17] Illoldi-Rangel P., Ciarleglio M., Sheinvar L., Linaje M., Sánchez-Cordero V., Sarkar S. (2012). Opuntia in México: Identifying Priority Areas for Conserving Biodiversity in a Multi-Use Landscape. PLoS ONE.

[18] Zou D., Brewer M., Garcia F., Feugang J.M., Wang J., Zang R., Zou C. (2005). Cactus pear: A natural product in cancer chemoprevention. Nutr. J..

[19] Zorgui L., Ayed-Boussema I., Ayed Y., Bacha H., Hassen W. (2009). The antigenotoxic activities of cactus (*Opuntia ficus-indica*) cladodes against the mycotoxin zearalenone in Balb/c mice: Prevention of micronuclei, chromosome aberrations and DNA fragmentation. Food Chem. Toxicol..

[20] Serra A.T., Poejo J., Matias A.A., Bronze M.R., Duarte C.M.M. (2013). Evaluation of *Opuntia* spp. derived products as antiproliferative agents in human colon cancer cell line (HT29). Food Res. Int..

[21] Díaz Medina E.M., Rodríguez Rodríguez E.M., Díaz Romero C. (2007). Chemical characterization of Opuntia dillenii and *Opuntia ficus indica* fruits. Food Chem..

[22] Moussa-Ayoub T.E., El-Samahy S.K., Rohn S., Kroh L.W. (2011). Flavonols, betacyanins content and antioxidant activity of cactus *Opuntia macrorhiza* fruits. Food Res. Int..

[23] Yahia E.M., Mondragon-Jacobo C. (2011). Nutritional components and anti-oxidant capacity of ten cultivars and lines of cactus pear fruit (*Opuntia* spp.). Food Res. Int..

[24] Coria Cayupán Y.S., Ochoa M.J., Nazareno M.A. (2011). Health-promoting substances and antioxidant properties of *Opuntia* sp. fruits. Changes in bioactive-compound contents during ripening process. Food Chem..

[25] Kuti J.O. (2004). Antioxidant compounds from four *Opuntia cactus* pear fruit varieties. Food Chem..

[26] Sumaya-Martínez M.T., Cruz-Jaime S., Madrigal-Santillán E., García-Paredes J.D., Cariño-Cortés R., Cruz-Cansino N., Alanís-García E. (2011). Betalain, Acid Ascorbic, Phenolic Contents and Antioxidant Properties of Purple, Red, Yellow and White Cactus Pears. Int. J. Mol. Sci..

[27] Kaur M. (2012). Pharmacological actions of *Opuntia ficus indica*: A Review. J. App. Pharm. Sci..

[28] Madrigal-Santillán E., García-Melo F., Morales-González J., Vázquez-Alvarado P., Muñoz-Juárez S., Zuñiga-Pérez C., Hernández-Ceruelos A. (2013). Antioxidant and Anticlastogenic Capacity of Prickly Pear Juice. Nutrients.

[29] Alimi H., Hfaeidh N., Bouoni Z., Sakly M., Ben Rhouma K. (2012). Protective effect of *Opuntia ficus indica* f. inermis prickly pear juice upon ethanol-induced damages in rat erythrocytes. Alcohol.

[30] Kim S.H., Jeon B.J., Kim D.H., Kim T.I., Lee H.K., Han D.S., Sung S.H. (2012). Prickly Pear Cactus (*Opuntia ficus indica* var. saboten) Protects Against Stress-Induced Acute Gastric Lesions in Rats. J. Med. Food.

[31] Wolfram R., Budinsky A., Efthimiou Y., Stomatopoulos J., Oguogho A., Sinzinger H. (2003). Daily prickly pear consumption improves platelet function. Prostaglandins Leukot. Essent. Fat. Acids.

[32] Jaeschke H., Williams C.D., McGill M.R., Xie Y., Ramachandran A. (2013). Models of drug-induced liver injury for evaluation of phytotherapeutics and other natural products. Food Chem. Toxicol..

[33] Jia Z., Tang M., Wu J. (1999). The determination of flavonoid contents in mulberry and their scavenging effects on superoxide radicals. Food Chem..

[34] Stintzing F.C., Herbach K.M., Mosshammer M.R., Carle R., Yi W., Sellappan S., Akoh C.C., Bunch R., Felker P. (2005). Color, Betalain Pattern, and Antioxidant Properties of Cactus Pear (*Opuntia* spp.) Clones. J. Agric. Food Chem..

[35] Dürüst N., Sümengen D., Dürüst Y. (1997). Ascorbic acid and element contents of foods of Trabzon (Turkey). J. Agric. Food Chem..

[36] Georgé S., Brat P., Alter P., Amiot M.J. (2005). Rapid Determination of Polyphenols and Vitamin C in Plant-Derived Products. J. Agric. Food Chem..

[37] Morales F.J., Jiménez-Pérez S. (2001). Free radical scavenging capacity of Maillard reaction products as related to colour and fluorescence. Food Chem..

[38] Re R., Pellegrini N., Proteggente A., Pannala A., Yang M., Rice-Evans C. (1999). Antioxidant activity applaying an improved ABTS radicalcation decolorization assay. Free Radic. Biol. Med..

[39] Kuskoski E.M., Asuero A.G., García-Parilla M.C., Troncoso A.M., Fett R. (2004). Actividad antioxidante de pigmentos antociánicos. Braz. J. Food Technol..

[40] Hinneburg I., Damien Dorman H.J., Hiltunen R. (2006). Antioxidant activities of extracts from selected culinary herbs and spices. Food Chem..

[41] Gulcin İ., Buyukokuroglu M.E., Kufrevioglu O.I. (2003). Metal chelating and hydrogen peroxide scavenging effects of melatonin. J. Pineal Res..

[42] Huang D., Ou B., Hampsch-Woodill M., Flanagan J.A., Prior R.L. (2002). High-Throughput Assay of Oxygen Radical Absorbance Capacity (ORAC) Using a Multichannel Liquid Handling System Coupled with a Microplate Fluorescence Reader in 96-Well Format. J. Agric. Food Chem..

[43] Conde de la Rosa L., Schoemaker M.H., Vrenken T.E., Buist-Homan M., Havinga R., Jansen P.L.M., Moshage H. (2006). Superoxide anions and hydrogen peroxide induce hepatocyte death by different mechanisms: Involvement of JNK and ERK MAP kinases. J. Hepatol..

[44] Vrenken T.E., Buist-Homan M., Kalsbeek A.J., Faber K.N., Moshage H. (2008). The active metabolite of leflunomide, A77 1726, protects rat hepatocytes against bile acid-induced apoptosis. J. Hepatol..

[45] Zhou G., Chen Y., Liu S., Yao X., Wang Y. (2013). In vitro and in vivo hepatoprotective and antioxidant activity of ethanolic extract from *Meconopsis integrifolia* (Maxim.) Franch. J. Ethnopharmacol..

[46] Hissin P.J., Hilf R. (1976). A fluorometric method for determination of oxidized and reduced glutathione in tissues. Anal. Biochem..

[47] Maeda K., Kimura M., Hayashi S. (1993). Cellular mechanism of U78517F in the protection of porcine coronary artery endothelial cells from oxygen radical-induced damage. Br. J. Pharmacol..

[48] Woudenberg-Vrenken T.E., Buist-Homan M., Conde de la Rosa L., Faber K.N., Moshage H. (2010). Anti-oxidants do not prevent bile acid-induced cell death in rat hepatocytes. Liver Int..

[49] Gu J., Gui Y., Chen L., Yuan G., Lu H.-Z., Xu X. (2013). Use of Natural Products as Chemical Library for Drug Discovery and Network Pharmacology. PLoS ONE.

[50] Bankova V. (2005). Chemical diversity of propolis and the problem of standardization. J. Ethnopharmacol..

[51] Kujumgiev A., Tsvetkova I., Serkedjieva Y., Bankova V., Christov R., Popov S. (1999). Antibacterial, antifungal and antiviral activity of propolis of different geographic origin. J. Ethnopharmacol..

[52] Anderson E.F. (2001). The Cactus Family.

[53] Vinson J.A., Su X., Zubik L., Bose P. (2001). Phenol antioxidants quantity and quality in foods: Fruits. J. Agric. Food Chem..

[54] Van Acker S.A., de Groot M.J., van den Berg D.-J., Tromp M.N., Donné-Op den Kelder G., van der Vijgh W.J., Bast A. (1996). A quantum chemical explanation of the antioxidant activity of flavonoids. Chem. Res. Toxicol..

[55] Nimse S.B., Pal D. (2015). Free radicals, natural antioxidants, and their reaction mechanisms. RSC Adv..

[56] Russo M., Spagnuolo C., Tedesco I., Bilotto S., Russo G.L. (2012). The flavonoid quercetin in disease prevention and therapy: Facts and fancies. Biochem. Pharmacol..

[57] Kumar S., Pandey A.K. (2013). Chemistry and Biological Activities of Flavonoids: An Overview. Sci. World J..

[58] Pan M.-H., Lai C.-S., Ho C.-T. (2010). Anti-inflammatory activity of natural dietary flavonoids. Food Funct..

[59] Frémont L., Gozzélino M.T., Franchi M.P., Linard A. (1998). Dietary flavonoids reduce lipid peroxidation in rats fed polyunsaturated or monounsaturated fat diets. J. Nutr..

[60] Fouad A.A., Albuali W.H., Zahran A., Gomaa W. (2014). Protective effect of naringenin against gentamicin-induced nephrotoxicity in rats. Environ. Toxicol. Pharmacol..

[61] Yokozawa T., Dong E., Kawai Y., Gemba M., Shimizu M. (1999). Protective effects of some flavonoids on the renal cellular membrane. Exp. Toxicol. Pathol..

[62] Nakayama M., Aihara M., Chen Y.-N., Araie M., Tomita-Yokotani K., Iwashina T. (2011). Neuroprotective effects of flavonoids on hypoxia-, glutamate-, and oxidative stress-induced retinal ganglion cell death. Mol. Vis..

[63] Wu Y., Wang F., Zheng Q., Lu L., Yao H., Zhou C., Wu X., Zhao Y. (2006). Hepatoprotective effect of total flavonoids from *Laggera alata* against carbon tetrachloride-induced injury in primary cultured neonatal rat hepatocytes and in rats with hepatic damage. J. Biomed. Sci..

[64] Pandey K.B., Rizvi S.I. (2009). Plant polyphenols as dietary antioxidants in human health and disease. Oxid. Med. Cell. Longev..

[65] Castellar R., Obón J.M., Alacid M., Fernández-López J.A. (2003). Color Properties and Stability of Betacyanins from *Opuntia* Fruits. J. Agric. Food Chem..

[66] Gandía-Herrero F., Escribano J., García-Carmona F. (2016). Biological activities of plant pigments betalains. Crit. Rev. Food Sci. Nutr..

[67] Livrea M.A., Tesoriere L., Neelwarne B. (2013). Lipoperoxyl Radical Scavenging and Antioxidative Effects of Red Beet Pigments. Red Beet Biotechnology: Food and Pharmaceutical Applications.

[68] Wu L., Hsu H.-W., Chen Y.-C., Chiu C.-C., Lin Y.-I., Ho J.A. (2006). Antioxidant and antiproliferative activities of red pitaya. Food Chem..

[69] Han J., Ma D., Zhang M., Yang X., Tan D. (2015). Natural antioxidant betanin protects rats from paraquat—induced acute lung injury interstitial pneumonia. Biomed. Res. Int..

[70] Madrigal-Bujaidar E., Álvarez-González I., Sumaya-Martínez M.T., Gutiérrez-Salinas J., Bautista M., Morales-González Á., González-Rubio M.G.Y., Aguilar-Faisal J.L., Morales-González J.A. (2014). Review of natural products with hepatoprotective effects. World J. Gastroenterol..

[71] Kanner J., Harel S., Granit R. (2001). Betalains, a new class of dietary cationized antioxidants. J. Agric. Food Chem..

[72] Li S., Tan H., Wang N., Zhang Z., Lao L., Wong C., Feng Y. (2015). The role of oxidative stress and antioxidants in liver diseases. Int. J. Mol. Sci..

[73] Abdelmegeed M.A., Moon K.-H., Chen C., Gonzalez F.J., Song B.-J. (2010). Role of cytochrome P450 2E1 in protein nitration and ubiquitin-mediated degradation during acetaminophen toxicity. Biochem. Pharmacol..

